# Molecular Dynamics Simulations Suggest Folded Chain Ends Compatible with the Cellulose-II Fibril Structure

**DOI:** 10.1021/acs.biomac.6c00983

**Published:** 2026-08-05

**Authors:** Linghan Kong, Stephen J. Eichhorn, Richard A. Bryce

**Affiliations:** † Division of Pharmacy and Optometry, 5292 University of Manchester, Oxford Road, Manchester M13 9PT, United Kingdom; ‡ Bristol Composites Institute, School of Civil, Aerospace and Design Engineering, Faculty of Science and Engineering, University of Bristol, Bristol BS8 1TR, United Kingdom; § School of Chemistry, Cantock’s Close, University of Bristol, Bristol BS8 1TS, United Kingdom

## Abstract

The structural feasibility of chain folding within an accepted cellulose-II structure is investigated in this study. We incorporate 3-residue and 5-residue folded glucosyl turns into a cellulose-II fibril crystal model to assess if the dominant structure can be retained. Ten candidate turn models were generated by metadynamics simulations and ranked by semiempirical quantum mechanical energies. Across >30 independent 300 ns molecular dynamics trajectories, the crystalline core retains its unit cell parameters, X-ray diffraction pattern, hydrogen bonding network, and canonical ^4^C_1_ ring conformation. This suggests that, should such folds be present, they need not compromise bulk crystalline integrity. No significant fibril twisting was observed in either the unfolded or chain-fold models. At the chain-folding ends of the fibril, high-energy boat and skew-boat conformations drive hydroxymethyl rotamer redistribution and noncanonical hydrogen bonding contacts. The greater structural flexibility of the 5-residue folded turns allows the chain-fold ends to access a broader conformational space, yet the structural perturbation remains localized within the same 3-residue region as the 3-residue folded turns. The 3-residue turn is therefore considered the more plausible chain-fold structure for cellulose-II, should such a structure actually exist in reality.

## Graphical abstract

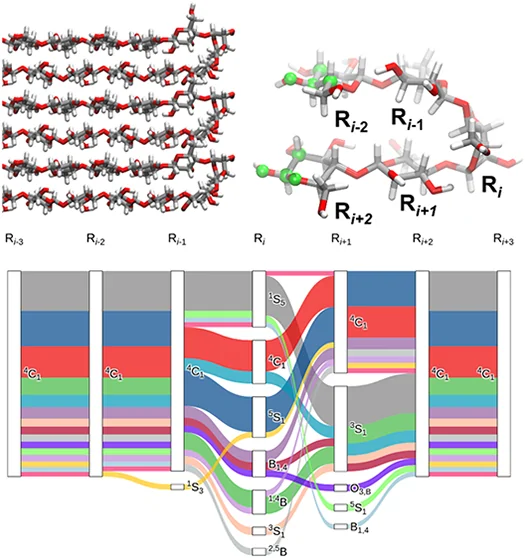

## Introduction

Cellulose is the most abundant naturally available polymeric material on the planet, and its use in society as a functional material has deep roots in our history.^1^ Cellulose is also one of the foundational structures that initiated our concepts of polymers, with the very early work of Staudinger using it as a premise for long chains of carbons, but also as a structure to interrogate other complex biopolymers.^2^ The concepts of cellulose being chain folded go back to an understanding of the spherulitic structure of so-called conventional oil-based polymers. These include polyethylene terephthalate^3^ and polyethylene,^4^ in pioneering studies by the Bristol physicist Andrew Keller, and the periodicity of “threads” pulled from these spherulitic structures.

Drawing on these observations, and electron microscopy images of apparent periodicity in cellulose fibrils, the Canadian-Jamaican scientist Rockcliffe St.J. Manley proposed a chain folded structure for native cellulose (cellulose I) in the 1960s.^5^ This idea of a chain folded structure was dismissed by Mark et al. in the late 1960s on the basis that such a structure would be too compliant.^6^ This being in contradiction with the very high crystal moduli (138 GPa) of cellulose that had been measured by Sakurada et al.^7^ Later, a packing study by the same group proposed that although Manley’s model violated the rule of mixtures, and would not result in an adequate stiffness to their material, a higher packing fraction model could be mechanically equivalent to the more acceptable extended chain model.^8^

Chain folded structures for cellulose-II are much more accepted in the literature. Indeed, a native form of cellulose II, produced by a mutant strain of *Acetobacter xylinum*, has been proposed to have a folded chain structure.^9^ This structure is thought to form because of the lack of a complex enzyme system for elementary fibril formation in bacterial and algal forms of cellulose.^10^ There are some other forms of bacterial cellulose, grown in constrained situations with another polymer, that demonstrate a chain folded structure.^11^ Some primitive algae are also known to exhibit chain folding in their cellulose chains.^10^ In these algae, however, yet another mechanism for folding occurs due to the lack of terminal complexes in their synthesis machinery leading to relatively disorganized growth.^10^ It is noted that periodic structures in cellulose II samples had been observed by Fischer and others (including Manley), although they stopped short of suggesting this was due to chain folding.^12^ Evidence for chain folding in cellulose recrystallized from dissolved material in hydrazine was not conclusive, but was proposed as a possible structure.^13^ It is quite clear that chain folding can occur in cellulose structures, but its occurrence may differ in respect of the reasons why it appears at all. In synthetic polymers, such as polyethylene, large single crystals are formed from solution wherein the chains are orthogonal to the plane of the crystal;^4^ this being discovered in 1957 by Andrew Keller. The notion of chain folding of cellulose has been discussed with reference to these synthetic polymers.^14^ In a discursive paper by Lindenmeyer it was argued that cellulose may contain chain folds, and this might be the source of the antiparallelism of adjacent chains.^14^ Also noted is that the most thermodynamically stable form of cellulose chains is an extended conformation.^14^ So it would seem rare that cellulose would adopt folded chains, unless constrained in some way, or because of molecular weight considerations.

One of the paradoxes of the mercerization of cellulose, where native plant material is treated with sodium hydroxide, is that the structure that forms (seemingly without dissolution) is thought to be cellulose-II. A feature of cellulose-II is that it has an antiparallel chain structure, where the polarities of the chains are reversed with respect to each other; cellulose-I is found to possess a parallel chain structure. Many theories as to why this occurs have been proposed in the literature. One theory, suggested by Kim et al.,^15^ proposed an intermingling of proximal chains of cellulose during mercerization to give rise to this antiparallel structure. This was evidenced by the gold labeling of the reducing ends of chains after reductive amination. Some have even suggested that cellulose-II might have a parallel chain conformation,^16^ although this idea is not widely accepted.

A number of modeling studies of chain folded structures have been published. One early study, in 2000, showed, using molecular dynamics, that chain folding is possible.^17^ This study however recognized that in a larger structure there would be constraints on this occurring.^17^ Notable from this study was that the glucose rings did not form “boat” type conformations in making folds, and so they were perhaps not tight enough for realistic crystals.^17^ Also, the 2-fold screw axis of a cellulose chain was not preserved in their optimized structures.^17^

Yamane et al.^18^ suggested that a folded chain structure ought to be possible for cellulose-II, but not for cellulose-I, based on modeling a sheet of cellulose with imposed lateral periodicity. Johnson and French showed, from molecular models, that certain geometries for cellulose produced significant helical cavities, and suggested these for the shapes of folds in a cellulose chain.^19^ Other work by Tanaka and Fukui^20^ has suggested that only very dilute systems of cellulose chains, in water, could produce folds, although French was critical of this study with respect to the realities of cellulose structures in aqueous environments.^21^

A more recent publication by Sawada et al.^22^ has proposed an experimentally verified chain folded structure for a mercerized bacterial cellulose sample i.e. cellulose II. Interestingly this proposition is confirmed by neutron diffraction, almost identical to the experiments carried out by Fischer et al.,^12^ but notably on a native cellulose structure that is not from a plant origin—so perhaps predicated to chain folding. Nevertheless, such structures deserve greater interrogation from a fundamental point of view.

Herein, we model a cellulose-II fibril (6 × 6 cross-section) using the GLYCAM06j force field,^23^ solvated in explicit water.^24^ The use of a fibril structure rather than a periodically constrained single-layer cellulose sheet^18^ provides a more realistic assessment of the propensity of cellulose to form chain folds that are compatible with the cellulose-II structure. We systematically investigate the conformational landscape of chain folding by constructing distinct models of 3-residue and 5-residue turn fragments. These folded oligosaccharide structures were initially generated using metadynamics simulations with the GFN-FF force field^25^ to identify representative pucker combinations before being integrated into the fibril model. Across 33 independent 300 ns molecular dynamics (MD) trajectories of folded and nonfolded fibril systems with explicit water, we assess the structural stability of the folded-chain fibrils through analysis of unit cell parameters, simulated X-ray diffraction patterns, and hydrogen bonding networks. This suggests that, should such folds be present, the crystalline core need not be disrupted by their presence.

We further record the ring pucker conformations at the chain-fold ends, where boat and skew-boat states dominate at the turn residue, with distortions propagating to the adjacent residues in the form of noncanonical puckering states. Finally, we trace the pucker transition pathways across models initialized with distinct puckering combinations, identifying convergence toward a limited set of preferred conformational channels at the chain end region. We note that this study does not address the question of whether chain-folded cellulose-II structures form or represent thermodynamically preferred states; rather, our aim is to assess whether, if such folds were present, they would be structurally compatible with the known cellulose-II chain arrangement.

## Methods

To investigate the potential for folded edges in a cellulose-II fibril, we first built two types of short cellulose fragments containing three and five glucosyl residues (denoted **3mer** and **5mer** respectively), to represent the chain end region (see Figure 1a). The assumption that the turn would encompass 3- to 5-residues would seem reasonable, given earlier work by Yamane et al. but also more recent carbohydrate foldamer studies.^26^ The residue positions in these two turns are labeled *R*_*i*‑1_ to *R*_*i*+1_ for the **3mer** and *R*_*i*‑2_ to *R*_*i*+2_ for the **5mer** (Figure 1a). These fragments were initially manually folded and adjusted to bridge the connecting interatomic distance between two antiparallel chains in a cellulose-II lattice. Specifically, two adjacent antiparallel chains were constructed using Cellulose-Builder.^27^ These were where the C1 atom of the terminal residue (for **3mer**) or penultimate residue (for **5mer**) at the nonreducing end of the origin chain and the O4 atom of the first (or second) residue at the reducing end of the center chain selected as anchor sites for subsequent structural modification. The distance between these two anchor sites was measured as 4.46 Å for accommodating the **3mer** model and 5.29 Å for the **5mer** model (Figure 1a). The end-to-end distance between the corresponding C1 and the O4 atoms of the folded turn fragments was then adjusted to match these values.

**Figure 1:**
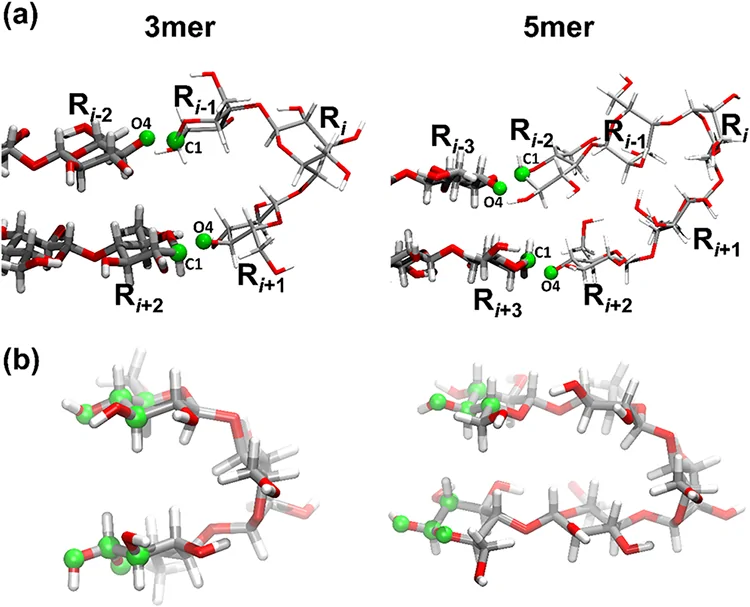
(a) Schematic representation of **3mer** (left) and **5mer** (right) turn fragments bridging two antiparallel cellulose chains, with residue positions labeled *R*_*i*‑2_ to *R*_*i*+2_ (**3mer**) and *R*_*i*‑3_ to *R*_*i*+3_ (**5mer**), where *R*_*i*_ represents the residue that facilitates the turn. Green spheres indicate the C1 and O4 anchor atoms used to define end-to-end distance constraints of 4.46 Å (**3mer**) and 5.29 Å (**5mer**), respectively. (b) Representative structures of the **3mer** (left) and **5mer** (right) turn fragments generated from metadynamics conformational exploration using GFN-FF force field, with harmonic positional restraints applied to anchor atoms in green.

Conformational exploration of the **3mer** and **5mer** models was then performed using the CREST toolkit^28^ to identify energetically favorable and geometrically feasible turn structures. To do this harmonic positional restraints with a force constant of 0.5 E_h_/a_0_^2^ (i.e., ∼1120 kcal/mol/Å^2^) were applied to the aforementioned anchor atoms and their immediate neighboring atoms to maintain compatibility with the crystal geometry (Figure 1b). It should be noted therefore that this study does not consider the spontaneous formation of folds, but rather that they are added to the system as a test of compatibility. Metadynamics simulations with the GFN-FF force field were carried out with these restraints, followed by calculation of single point energies via the semiempirical quantum mechanics method GFN2-xTB.^29^ The conformational analyses employed CREST’s automated metadynamics sampling scheme.^30^ Convergence was ensured by CREST’s iterative workflow, in which metadynamics and genetic crossing cycles are repeated whenever a new lowest-energy conformer is identified, terminating automatically once successive cycles fail to yield any further improvement in the global minimum. This procedure generated ensembles of 23,539 and 34,208 conformers for the **3mer** and **5mer** models, respectively, within an energy window of 15 kcal/mol. From the resulting ensembles, five representative structures for each fragment turn model (numbered **I** to **V**, Table 1) were manually selected for later integration into the 6 × 6 cellulose-II fibril crystal model (**CM**). To maximize structural diversity within the turn model, conformers were selected primarily to represent distinct ring pucker combinations, with additional representatives drawn from progressively higher relative energies once the lower-energy pucker types had been covered.

**Table 1:** Ring Pucker Conformations at Residue Positions *R*_*i*‑2_ to *R*_*i*+2_ and Relative GFN2-xTB Energies of the Five Representative **3mer** and **5mer** Turn Models (Numbered **I** to **V**) Selected from the Metadynamics Conformational Ensemble^a^

Model	*R*_*i*‑2_	*R*_*i*‑1_	*R*_*i*_	*R*_*i*+1_	*R*_*i*+2_	Δ*E*_GFN2‑xTB_ (kcal/mol)
**3mer-I**		^4^C_1_	^5^S_1_	^4^C_1_		0.0
**3mer-II**		^4^C_1_	B_1,4_	^4^C_1_		1.7
**3mer-III**		^O^S_2_	^2^S_O_	^4^C_1_		2.0
**3mer-IV**		^4^C_1_	^1^S_5_	^4^C_1_		8.9
**3mer-V**		^4^C_1_	^1,4^B	^4^C_1_		11.6
**5mer-I**	^4^C_1_	^1^C_4_	^1^S_5_	^4^C_1_	^4^C_1_	0.0
**5mer-II**	^4^C_1_	^1^C_4_	^1,4^B	^4^C_1_	^4^C_1_	0.0
**5mer-III**	^4^C_1_	^4^C_1_	^1,4^B	^4^C_1_	^4^C_1_	1.1
**5mer-IV**	^4^C_1_	^2^S_O_	^5^S_1_	^4^C_1_	^4^C_1_	10.4
**5mer-V**	^4^C_1_	B_3,O_	B_1,4_	^4^C_1_	^4^C_1_	11.7

a Energies referenced to the lowest-energy conformer within each model (**3mer-I** and **5mer-I**, respectively).

This cellulose-II fibril model **CM**, carved from a larger ideal cellulose-II supercell generated using the Cellulose-Builder program, consists of a fibril with 36 chains in a 6 × 6 layout, each chain comprising 40 glucose residues in length (Figure 2a,b). This model was modified to accommodate the selected folded fragments: from each cellulose chain, one residue (or two residues for **5mer**) was removed from both the reducing and nonreducing ends to maintain an equal number of residues in the long nonturn region of the chains. The folded fragments were then attached to both ends of the chains, thereby merging six antiparallel chains into a single folded chain and resulting in six folded chains arranged side-by-side within the cellulose-II crystal lattice (Figure 2c). In total, 11 fibril models were constructed: five **3mer** cellulose-II models, five **5mer** models and one reference **CM** cellulose-II model without folded fragments attached (i.e., with no linker between adjacent chains).

**Figure 2:**
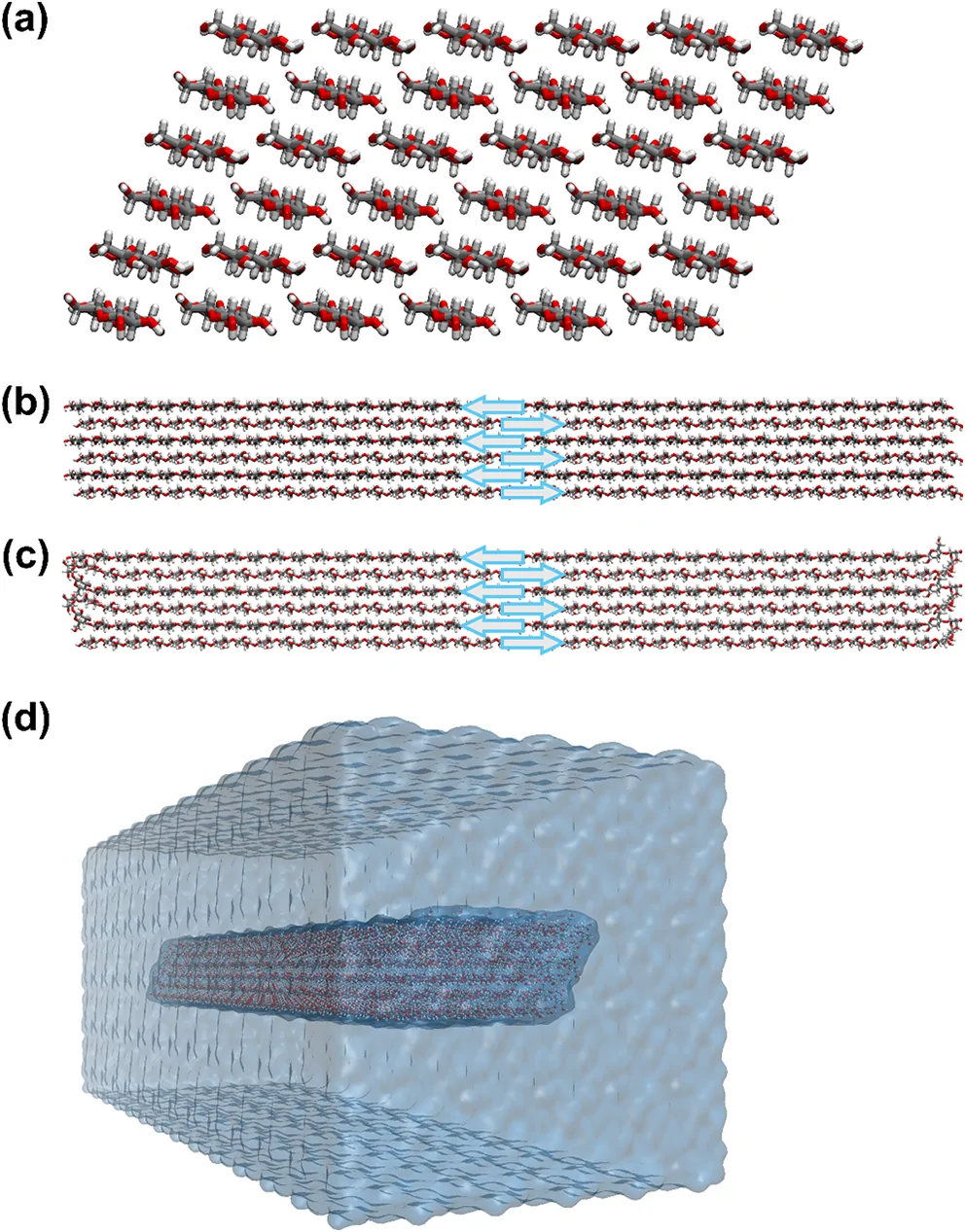
(a) Cross-sectional view of the 6 × 6 cellulose-II fibril crystal model (**CM**) comprising 36 chains of 40 glucose residues each, viewed along the fibril axis. (b) Side view of the unfolded **CM**, and (c) side view of the folded-chain model (**3mer**), with blue arrows indicating the chain direction from the reducing end to the nonreducing end. (d) The **CM** solvated in an explicit TIP3P water box (blue) of 100 Å × 100 Å × 250 Å, was used as a basis for all molecular dynamics (MD) simulations.

Molecular dynamics (MD) were performed using the Amber24 simulation package^31^ to assess the stability of the folding systems. The cellulose fibrils were modeled with the GLYCAM06j force field, and the structures were solvated in a TIP3P water box of 100 Å × 100 Å × 250 Å, resulting in a total of approximately 240,000 atoms (Figure 2d). This combination of solute and solvent force field is a widely deployed model in simulation of carbohydrates in solution. Previous simulation studies of cellulose-Iβ fibrils exploring the effect of force fields have demonstrated that GLYCAM06 can provide a reasonable description of the structure of these nanostructures in TIP3P water.^32^ However, it was observed that GLYCAM06 slightly underestimates the *c* lattice parameter of cellulose Iβ.^32^ We also note that considerable differences in pyranose ring inversion propensity have been observed as a function of different carbohydrate force fields.^33^ The water model, TIP3P, which is very widely used, similarly has known limitations, such as overestimated diffusion coefficients^34^ and underestimated levels of hydrogen bonding. These limitations should be borne in mind when considering the combination of solute and water models in this work.

A stepwise equilibration protocol was adopted to minimize unfavorable structural distortions during the construction and initial solvation phase. The systems first underwent energy minimization to relieve steric clashes, during which the nonturn regions (crystalline core) of the cellulose fibril were restrained with positional harmonic restraints of 100 kcal/mol/Å^2^. This was carried out together with torsional restraints on the endocyclic dihedral angles within the folded regions to preserve the initial ring pucker conformations. Each system was then heated from 0 to 298 K over 300 ps with these restraints and maintained at this temperature for an additional 200 ps in the NVT ensemble. This was followed by 500 ps of equilibration in the NVT ensemble and a subsequent 500 ps in the NPT ensemble at 1 bar with restraints maintained throughout. The force constants for both positional and dihedral restraints were then reduced to 10 kcal/mol/Å^2^ for a further 500 ps of NPT equilibration. Finally, all restraints were removed and the unrestrained system was allowed to equilibrate for 1.2 ns at the NPT ensemble at 298 K and 1 bar prior to production simulations.

To ensure sufficient sampling, for each of the 11 cellulose-II models built, three independent replicates of 300 ns MD simulations (labeled **a** to **c**) were performed in the NPT ensemble, starting with different assigned initial velocities. The temperature was maintained at 298 K using a Langevin thermostat^35–37^ with a collision frequency of 1.0 ps^–1^ and the pressure kept at 1 bar using a Monte Carlo barostat with isotropic scaling.^38^ The time step was set to 2 fs with the SHAKE algorithm^39^ applied to bonds involving hydrogen atoms. Short-range nonbonded interactions were truncated with a cutoff of 12 Å, and long-range Coulomb interactions were treated using the particle-mesh Ewald (PME) method.^40^ Trajectories were saved every 1 ns for subsequent analysis. Overall, 33 replicates of 300 ns MD simulations for 11 fibril systems were performed.

Following the MD simulations, trajectory analyses were performed to characterize the structural stability and conformational dynamics of the fibril models. The MD trajectories were analyzed using CPPTRAJ^41^ and MDAnalysis.^42,43^ Unit cell parameters were determined by selecting a central unit cell from the crystalline core and measuring its lattice vectors along the 300 ns MD trajectories. The lattice parameters were obtained by ensemble averaging across three independent replicates for the **CM** and across all folded models (**3mer** and **5mer**, 30 trajectories in total). The chain twist dihedral angle was defined as the O4_*n*_–O4_*n*+1_–O4_*n*+2_–O4_*n*+3_ dihedral along each cellulose chain in the crystalline core, where consecutive O4 atoms span two cellobiose units and the subscript *n* denotes the residue index counted from the reducing end of the chain. This dihedral was calculated for all eligible chains in the crystalline core along the 300 ns MD trajectories and the ensemble-averaged across all trajectories for each of the three models. Simulated X-ray diffraction (XRD) patterns for the cellulose-II fibril were computed using the Debyer program (https://github.com/wojdyr/debyer). The ideal cellulose-II diffraction pattern was generated by the Mercury program from the Cambridge Crystallographic Data Centre,^44^ from a CIF-format file obtained from a previous study.^45^ All XRD calculations were performed using a Cu Kα wavelength of 1.541 Å and a step size of 0.05°. To account for disruption of periodic lattice planes at the fibril model surfaces, a reconstructed supercell approach was employed, in which the coordinates of the **3mer-I-a** replicate at 300 ns were embedded into the corresponding region of an ideal cellulose-II supercell prior to the diffraction calculation (inset, Figure 3).

**Figure 3:**
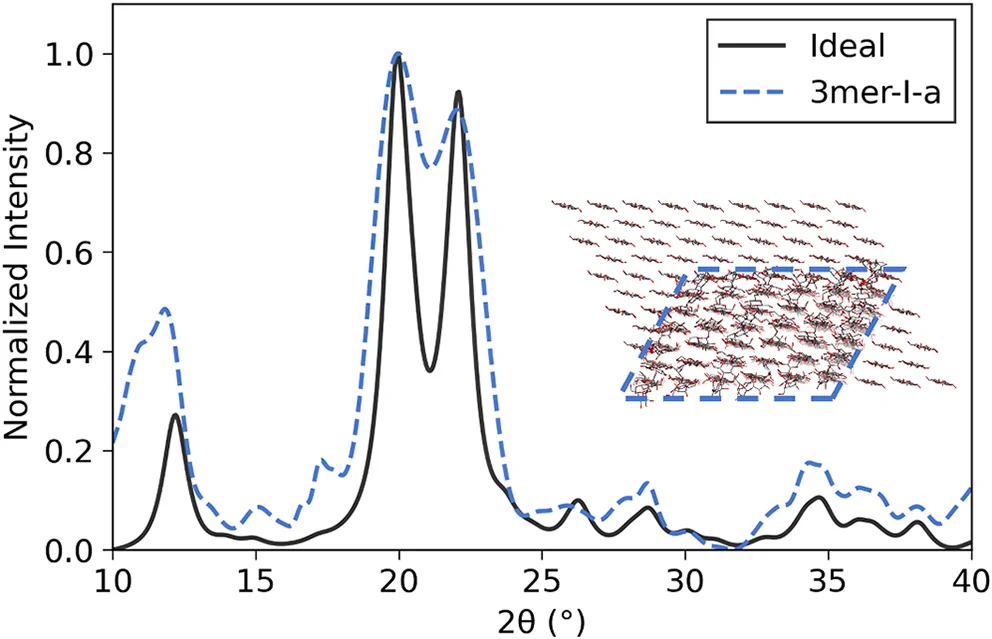
Simulated XRD patterns of the MD-grafted **3mer-I-a** structure at 300 ns (blue-dashed line) compared against the ideal cellulose-II reference (black solid line). The inset shows the reconstructed cellulose-II supercell with the region replaced by the 300 ns MD snapshot coordinates of **3mer-I-a** outlined in blue dashed box, while the surrounding region retains the ideal crystal structure.

## Results and Discussion

To evaluate the structural stability and validity of the simulated models of folded cellulose-II, unit cell parameters of the fibril crystalline core for both the **CM** and the folded models were compared against experimental cellulose-II crystallographic data^46^ (Table 2). The **CM** unit cell parameters show close agreement with experimental values, with deviations in lattice lengths within 5%. Similar difference between MD-simulated and experimentally determined unit cell parameters have been previously reported using the GROMOS r56Acarbo force field.^47^ Upon incorporation of the folded fragments into the model, a modest increase in unit cell dimensions was observed. These changes were predominantly in the *a* and *b* lattice parameters (0.8% and 0.5% respectively relative to the **CM**), while the chain-axis parameter *c* remained largely unchanged. This anisotropic expansion is consistent with the lattice strain associated with accommodating the folded chain conformation within the crystalline lattice.

**Table 2:** Comparison of Unit Cell Parameters of Cellulose-II Obtained from Experiment, the Unfolded Crystal Model (**CM**) Averaged Over 3 × 300 ns MD Trajectories, and the Folded Chain Models (**3mer** and **5mer**) Averaged over 30 × 300 ns MD Trajectories

Parameters	Experimental	Crystal Model	Folded Chain Models
*a* (Å)	8.10	8.40	8.47
*b* (Å)	9.03	8.77	8.81
*c* (Å)	10.31	10.73	10.73
γ (deg)	117.1	118.5	117.9

Next, we assessed the XRD pattern of the chain-fold model after a 300 ns MD simulation. The XRD pattern of the MD-grafted structure was in close agreement with that of an ideal cellulose-II crystal,^45^ reproducing the characteristic Bragg peaks corresponding to the (1–10), (110) and (020) planes (Figure 3). The corresponding peak positions in the folded-chain model were observed at 2θ = 11.83°, 19.98°, and 22.03°, in good agreement with the ideal cellulose-II reference peaks at 12.20°, 19.95°, and 22.10°, respectively (Table S2). After normalizing the strongest peak to 1.0, the relative intensities of these reflections were 0.52, 1.0, and 0.90 for the folded-chain model, compared with 0.28, 1.0, and 0.92 for the ideal cellulose-II reference (Table S2). The preservation of both peak positions and the dominant intensity pattern supports the possibility that the crystalline core of the folded-chain model is capable of retaining a cellulose-II lattice structure. The agreement of the predicted patterns with other diffraction data, particularly neutron diffraction data^22^ in the context of this study, does not imply chain folding, however, and is really only a demonstration of consistency. The broader peaks and elevated diffuse scattering observed across the pattern were attributed to the thermal disorder inherent to finite-temperature MD simulations and the local structural irregularities introduced by the amorphous chain end regions at the fibril surfaces.

To further assess the dynamical stability of the models in an explicit solvent environment, the mass-weighted all-atom root-mean-square deviation (RMSD) was calculated over the replicate trajectories of the cellulose-II fibril. This was conducted with the crystalline core region and the chain end regions (Figure 4a) analyzed separately. The crystalline core region comprises 1224 glucose residues for all models. The chain end regions of the **CM** comprise 216 glucose residues in total, while those of the folded models contain 246 residues. This reflected an additional 30 residues introduced by the five incorporated folded turns across the six folded chains. In the crystalline core region (Figure 4b, top), the reference **CM**, **3mer** and **5mer** models converged to comparable all-atom RMSD values of approximately 0.8–0.9 Å. The **3mer** and **5mer** models showed marginally higher RMSD values relative to the **CM**, though the difference did not appear significant. This therefore confirmed that the presence of chain folds does not compromise the structural integrity of the crystalline core.

**Figure 4:**
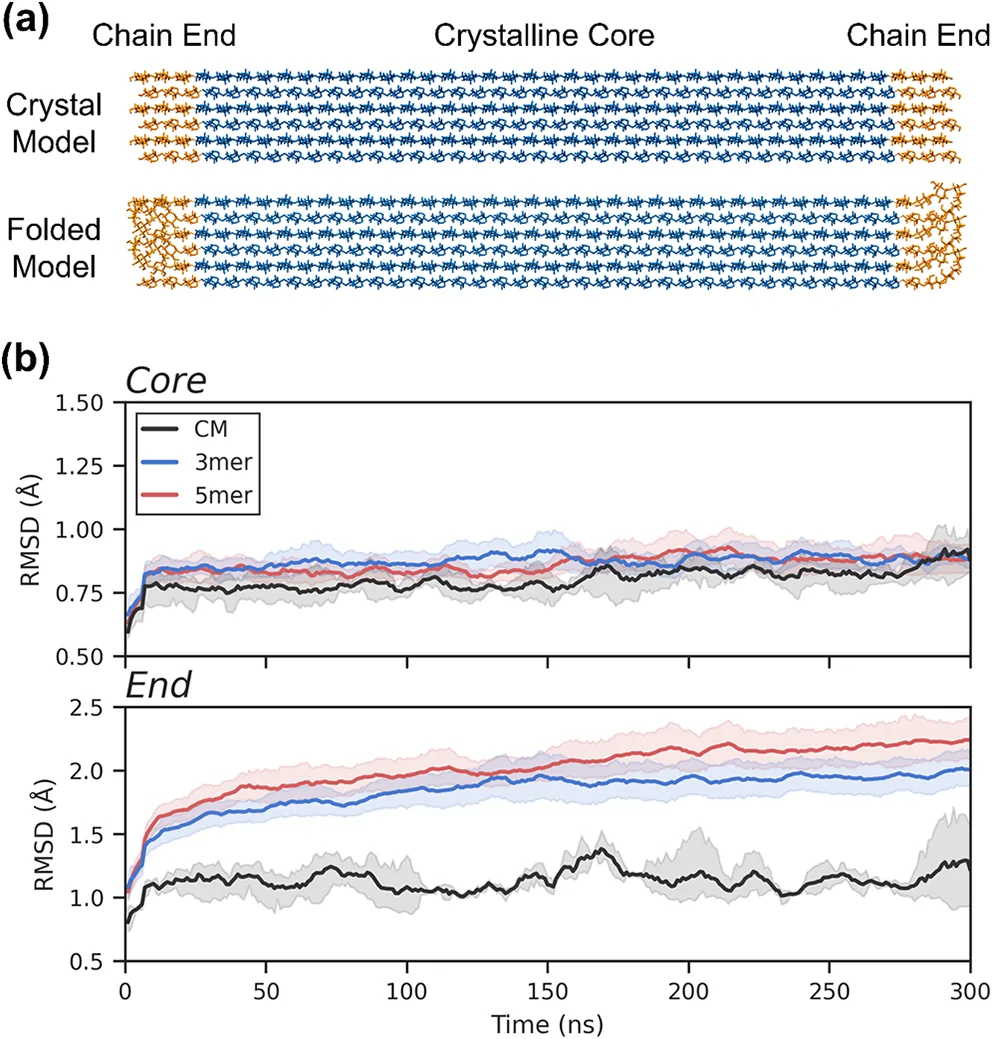
(a) Schematic representation of the unfolded crystal model (**CM**, top) and the folded chain model (bottom), with the crystalline core (blue) and chain end regions (orange) indicated. (b) Mass-weighted all-atom RMSD of the fibrils as a function of simulation time for the crystalline core (top) and chain end region (bottom) of the **CM** (black), **3mer** (blue), and **5mer** (red) models. For the **CM**, solid lines represent the mean over three independent replicates; for the **3mer** and **5mer** models, solid lines represent the mean over 15 replicates (three replicates across five models), with shaded areas denoting the corresponding standard deviation in all cases.

In the chain end regions (Figure 4b, bottom), the **CM** maintained a stable RMSD of approximately 1.0–1.3 Å throughout the simulation. The slightly higher RMSD values relative to the core region are expected, as the chain ends of the fibril are directly exposed to the surrounding solvent and therefore subject to reduced lattice constraints. By contrast, the **3mer** and **5mer** models showed substantially higher RMSD values, which increased progressively over the first ∼ 200 ns before plateauing at approximately 1.8 and 2.2 Å, respectively. We also observe that the ring pucker population in the chain end regions stabilized beyond 200 ns. Population distributions of the dominant pucker states remained consistent across the final 100 ns trajectories for both **3mer** and **5mer** models (Figure S1). This gradual rise followed by stabilization suggests an initial structural relaxation and conformational adjustment before reaching a stable conformation, rather than a disordering of the turns.

We note that no evidence of significant fibril twisting was observed in either the **CM** or chain-fold cellulose-II models over the course of the simulations, as characterized by the O4*_n_*–O4_*n*+1_–O4_*n*+2_–O4_*n*+3_ dihedral angle (Figure 5a). Across all three models, the average twist dihedral remains within a narrow range of approximately 180.0°–180.2° throughout the 300 ns trajectories, deviating only marginally from the ideal linear value of 180°. The **CM**, **3mer** and **5mer** models exhibit near-indistinguishable twist profiles, with overlapping standard deviations across the full simulation, indicating that the introduction of chain folds does not measurably alter fibril twist.

**Figure 5:**
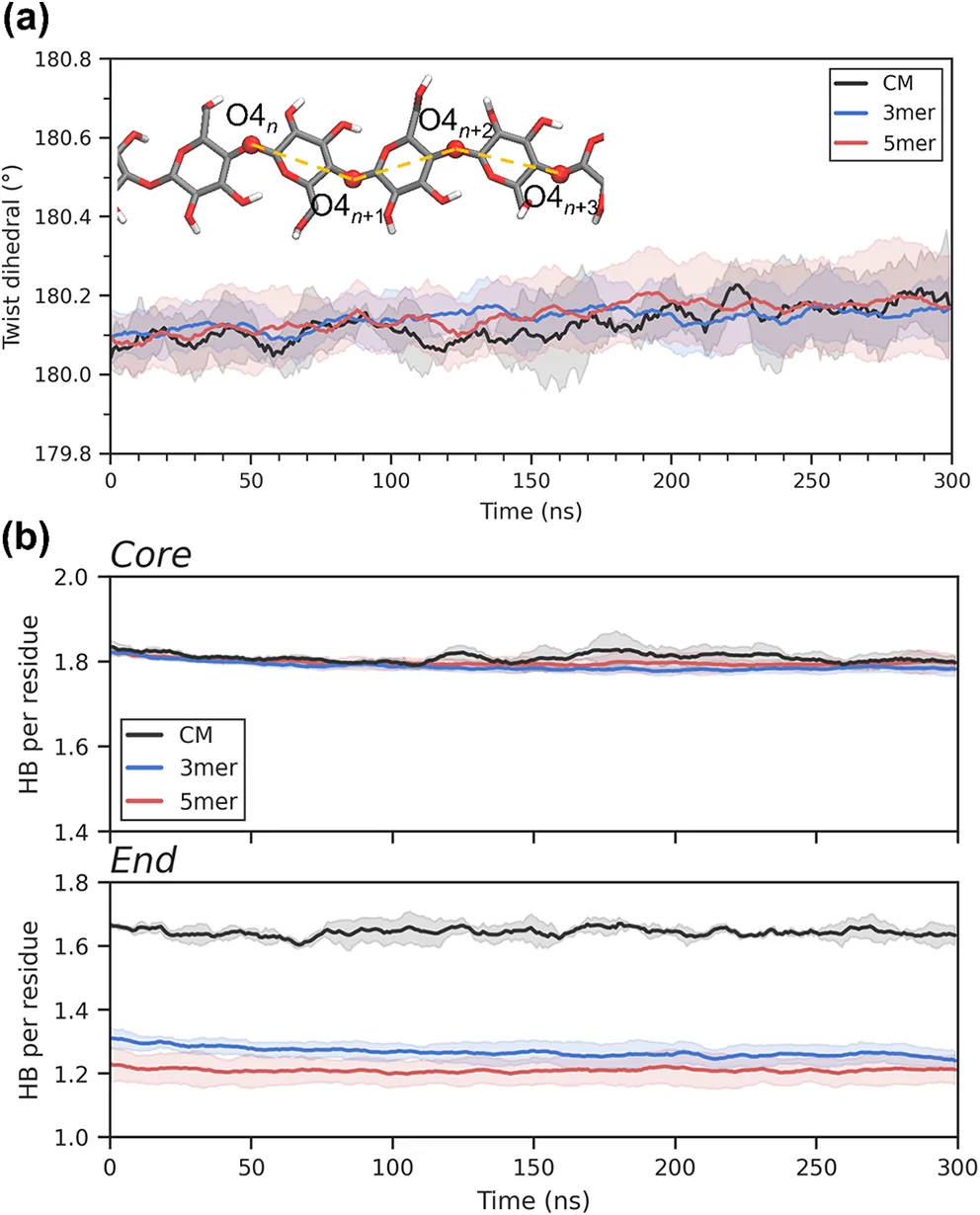
(a) Twist dihedral angle (O4_*n*_–O4_*n*+1_–O4_*n*+2_–O4_*n*+3_) of the cellulose-II nanofibril as a function of simulation time for the **CM** (black), **3mer** (blue) and **5mer** (red) models. Inset shows the four O4 atoms used to define the dihedral angle. (b) Hydrogen bond (HB) density per residue as a function of simulation time for the crystalline core (top) and chain end regions (bottom) of the **CM** (black), **3mer** (blue), and **5mer** (red) models. Averaging is performed as described in Figure 4, with shaded areas denoting the corresponding standard deviation in all cases.

This behavior contrasts with that reported for cellulose-Iβ nanofibrils, where a right-handed twist of approximately 2°/cellobiose has been observed in MD simulations.^48^ The structural stability of the models is supported by calculating the hydrogen bond density per residue calculated throughout the MD simulations (Figure 5b). In the crystalline core region, all three models maintain a similar hydrogen bond density of approximately 1.8 per residue across the entire simulation (Figure 5b, top). This indicates that the inclusion of chain folds does not significantly change the hydrogen bond density within the cellulose-II lattice. This observation is consistent with the RMSD results discussed above.

In the chain end regions, however, significant disparities were observed among the three models (Figure 5b, bottom). The **CM** reference maintains the highest hydrogen bond density in this region at approximately 1.6–1.7 per residue, reflecting the relatively ordered nature of the chain termini of the unfolded fibril. The **3mer** model shows a reduction of approximately 25% relative to its own crystalline core, stabilizing at around 1.3 hydrogen bonds per residue, while the **5mer** model exhibits a slightly greater reduction of approximately 30%, stabilizing at around 1.2 hydrogen bonds per residue. The difference in hydrogen bond density suggests that compared to **3mer** models, **5mer** models introduce a greater degree of local structural disruption at the fibril surfaces. This finding is further evidenced by the RMSD differences observed in the chain end region across the three models (Figure 4b, bottom).

To further characterize the hydrogen bonding network, the contributions of individual donor–acceptor (D–A) pairs were examined separately for the crystalline core and the chain end regions (Figure 6a, left). In the crystalline core, intrachain hydrogen bonding is dominated by the O3···O5 contact across all three models, accounting for approximately 99% of all intrachain hydrogen bonds in the **CM**, **3mer** and **5mer** models. Interchain hydrogen bonding is distributed across O2···O6, O6···O2, O2···O2 and O6···O6 pairs, accounting for approximately 30%, 25%, 23% and 21% of interchain contacts respectively, with near-identical distributions observed across all three models. Upon closer inspection, the interchain network is characterized by an interlayer hydrogen bonding pathway perpendicular to the chain planes. This follows the sequence ···O2c···O2o···O6o···O6c··· (Figure 6b), where subscripts c and o denote center and origin chains, respectively. Visual inspection of representative snapshots suggests that this interlayer pathway propagates throughout the fibril cross-section. This is consistent with findings reported in previous studies.^47,49,50^ The presence of this extensive interlayer hydrogen bonding network also provides a structural basis for the greater thermodynamic stability of cellulose-II relative to cellulose-Iβ,^51,52^ in which interlayer hydrogen bonding is largely absent.

**Figure 6:**
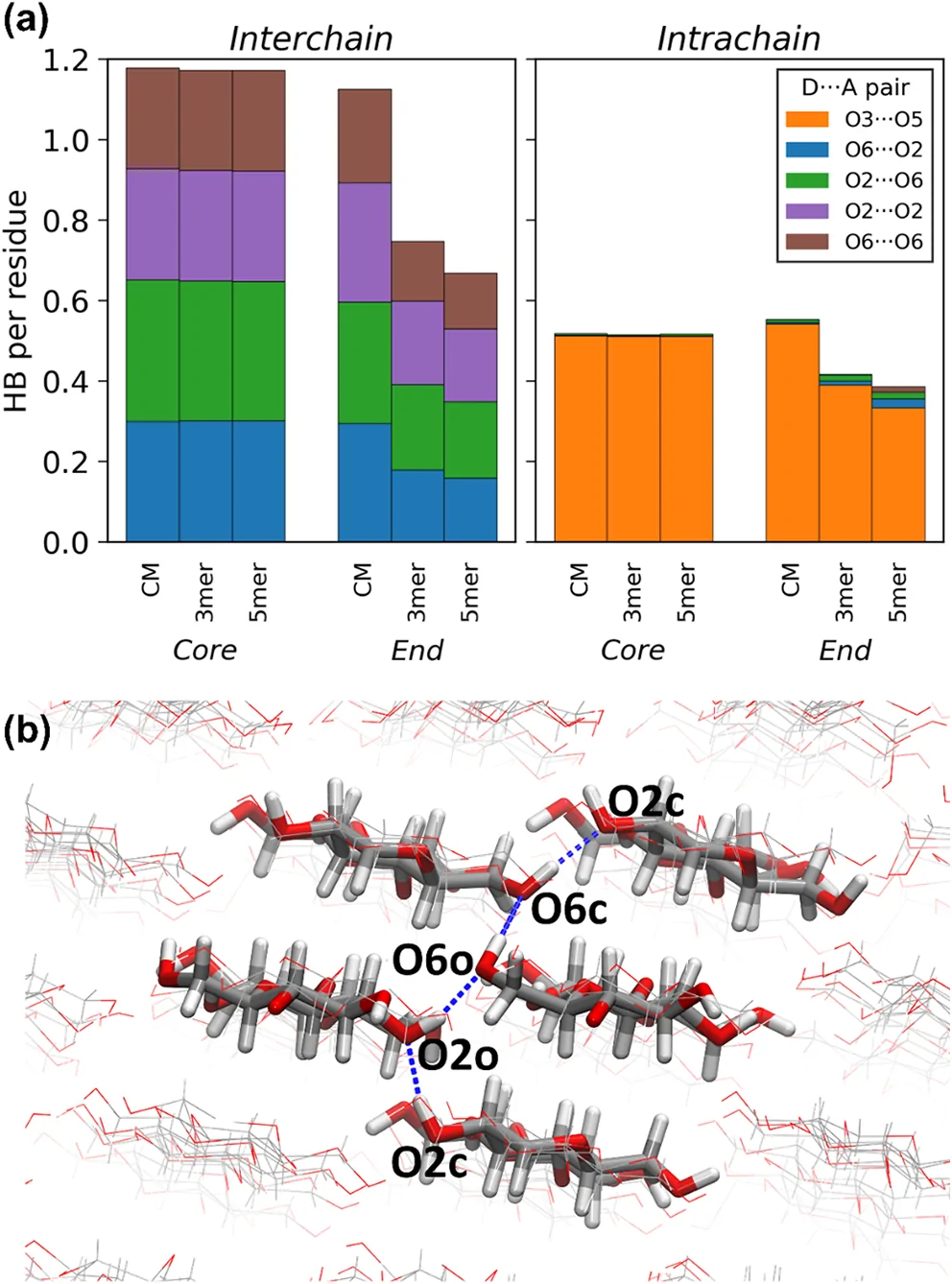
(a) Interchain (left) and intrachain (right) hydrogen bond density per residue decomposed by donor–acceptor (D–A) pair for the **CM**, **3mer** and **5mer** models, with crystalline core and chain end regions counted separately. (b) Representative snapshot illustrating the interlayer interchain hydrogen bonding pathway in the crystalline core, following the sequence ···O2c···O2o···O6o···O6c···, where subscripts c and o denote center and origin chains, respectively. Hydrogen bonds are indicated by blue dashed lines.

In the chain end regions, the intrachain hydrogen bonding pattern undergoes a significant redistribution relative to the crystalline core (Figure 6a, right). While the O3···O5 contacts remain the dominant intrachain interaction in the **CM** chain ends (98.1%), it is substantially reduced in the **3mer** (93.7%) and **5mer** (86.4%) models. The deficit is accounted for by a gain in noncanonical hydrogen bonds, i.e., not typically associated with interchain interactions in the crystalline core (Figure 6a, right). This redistribution is partially enabled by a conformational adaptation of the hydroxymethyl group (Figure 7a). In the crystalline core, the O5–C5–C6–O6 dihedral angle is predominantly populated in the *gt* conformation across all three models, with a secondary *gg* population (Figure 7a, top), consistent with the reported conformational preferences of cellulose-II.^18,53^ In the chain end regions, however, the *gt* population is considerably reduced in the **3mer** and **5mer** models relative to the **CM** reference, with the majority of this reduction accounted for by a gain in the *gg* conformation and a minor fraction transitioning to *tg* (Figure 7a, bottom). This shift in hydroxymethyl conformation likely relieves the steric strain imposed by the chain-fold geometry, and simultaneously reorients the hydroxymethyl group to satisfy the noncanonical intrachain hydrogen bonding described above (Figure 7b).

**Figure 7:**
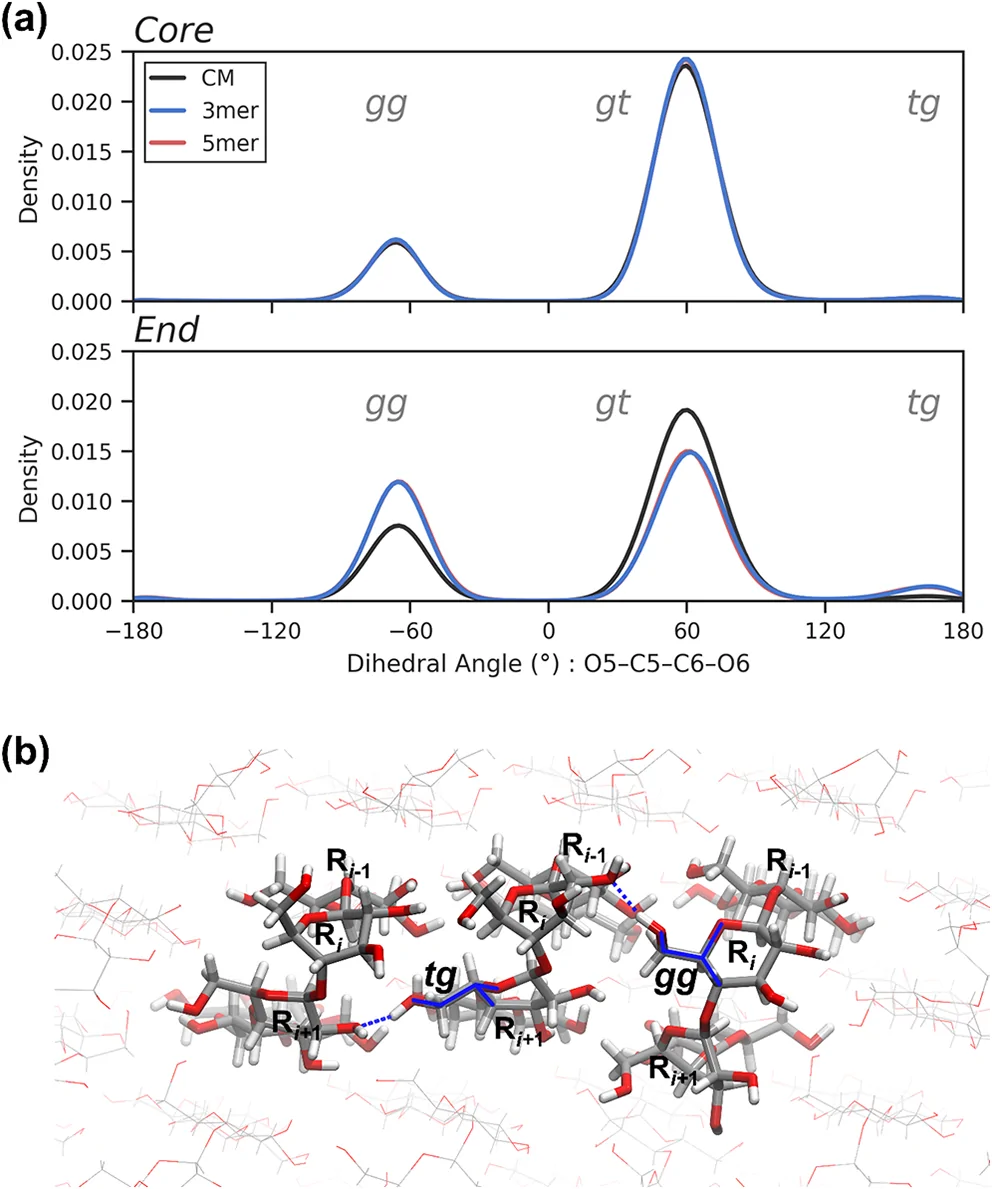
(a) Probability density distributions of the O5–C5–C6–O6 dihedral angle for the **CM** (black), **3mer** (blue), and **5mer** (red) models in the crystalline core (top) and chain end regions (bottom). (b) Representative snapshot of **3mer-I-a** at 300 ns illustrating noncanonical intrachain hydrogen bonding contacts (blue dashed lines) associated with the *tg* (left) and *gg* (right) hydroxymethyl conformations at the chain-fold end, with residue positions *R*_*i*‑1_, *R*_*i*_, and *R*_*i*+1_ labeled, with the dihedrals highlighted in blue.

The Cremer-Pople pyranose ring puckering conformations^54^ were analyzed for all models to assess the extent of ring distortion induced by chain folding (Figure 8). In the crystalline core region, all models maintain greater than 99% of residues in the canonical ^4^C_1_ chair conformation. In the chain end regions, however, significant conformational diversity is observed across both the **3mer** and **5mer** models, with 10–20% of the residues in these regions distributed among a range of strained boat, skew-boat and ^1^C_4_ conformers. This behavior is consistent with the folded-chain cellulose-II model proposed by Yamane et al., in which nonchair skew-boat puckers were required to accommodate the chain turn.^18^ However, whereas Yamane’s folded-turn model converged primarily to specific skew-boat states, namely ^5^S_1_ and ^1^S_5_, the present simulations reveal a broader distribution of strained puckering conformations in the corresponding chain end regions. This suggests that the folded models in this work explore a wider conformational landscape, while the ^4^C_1_ dominated crystalline core remains preserved. We also acknowledge that, even with multiple replicas, these 300 ns trajectories may be short for slow time scale sampling of ring pucker coordinates,^55^ or to observe large scale fibril reorganization. Nevertheless, these simulations probe a variety of folded fibril structures, and we find on these time scales, reorganization to stable structural minima, such that the pucker distribution of the final and penultimate 50 ns blocks converge (Figure S1).

**Figure 8:**
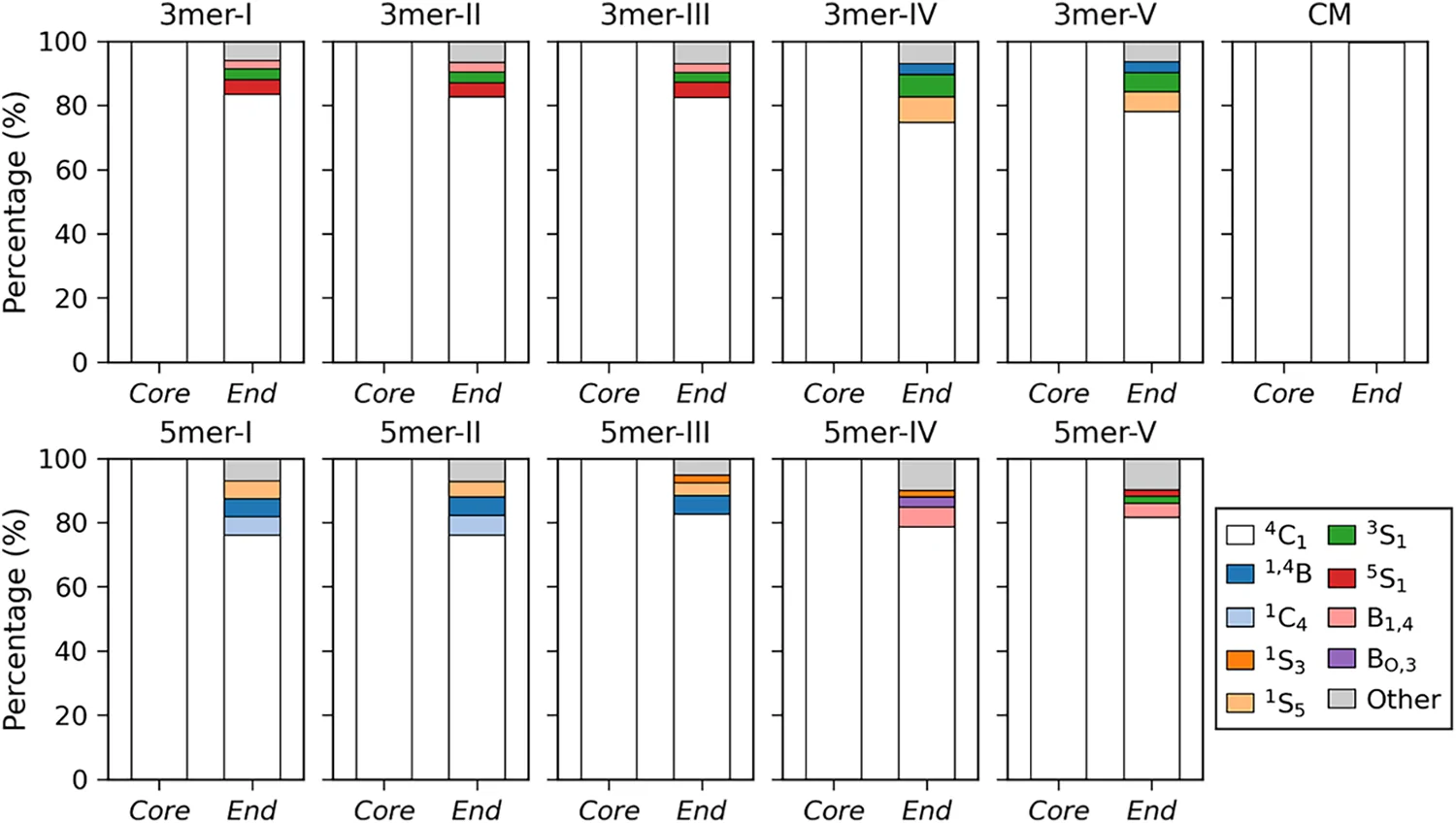
Pyranose ring pucker distributions for the crystalline core and chain end regions of each individual **3mer** (top) and **5mer** (bottom) model, alongside the **CM** reference. Bar charts show the percentage of residues adopting each puckering conformation, with the canonical ^4^C_1_ chair (white) and noncanonical boat, skew-boat, and ^1^C_4_ chair states indicated by the color legend.

Across the individual folded-chain fibril models, the pyranose rings did not always retain their initial puckering combinations throughout the 300 ns simulations. However, most folded chain fibril systems only undergo limited exploration of the Cremer-Pople conformational hypersurface, remaining confined to the vicinity of their initial puckering state rather than freely interconverting among all accessible conformations. This behavior suggests that the chain-fold geometry imposes a strong conformational bias on the pyranose rings at the fold turns, likely associated with substantial energy barriers between distinct puckering states. Nevertheless, the ensemble of models collectively samples a broad range of puckering combinations, demonstrating that multiple distinct puckering states can be stably maintained at the chain-fold turns over the 300 ns simulation time scale.

To characterize the propagation of ring distortion along the chain, the puckering conformations of residues at the folded turns were mapped as Sankey diagrams. These illustrate both the distribution of puckering states at each residue position and the transition pathways between adjacent positions (Figure 9). The top 15 most frequent puckering transition pathways are shown separately for the **3mer** and **5mer** models (Figure 9a,b).

**Figure 9:**
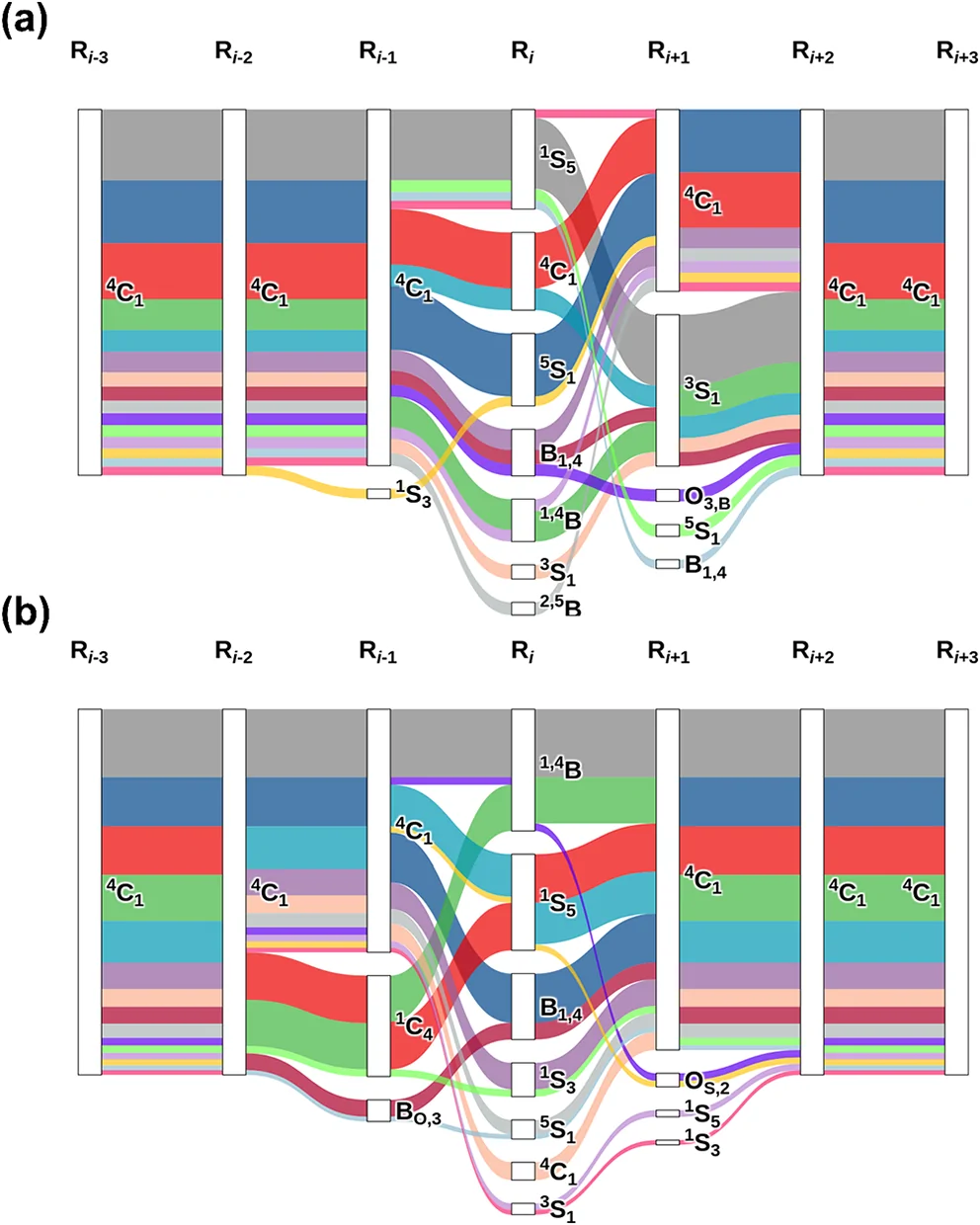
Sankey diagrams of distribution of pyranose ring puckering states and their transition pathways across residue positions *R*_*i*‑3_ to *R*_*i*+3_ at the chain end region of (a) **3mer** and (b) **5mer** models. The width of each band is proportional to the frequency of the corresponding puckering transition pathway, with the top 15 most probable pathways shown. Vertical bars represent the overall puckering state distribution at each residue position, normalized such that bar widths sum to unity.

For both models, ring distortion is localized to the three central residues *R*_*i*‑1_, *R*_*i*_ and *R*_*i*+1_, with residues beyond this region, i.e., *R*_*i*±2_, *R*_*i*±3_, etc., maintaining essentially purely ^4^C_1_ conformations. This would appear to confirm that the structural cost of chain folding is short ranged, confined to a depth of approximately three residues from the turn.^18^ At *R*_*i*_, high-energy boat and skew-boat conformations dominate, as required by the sharp geometric constraints of the chain folding. The distortion propagates outward to the immediately adjacent residues *R*_*i*–1_ and *R*_*i*+1_, though to a lesser extent. The two adjacent residues display asymmetric puckering distributions, reflecting the distinct local environments of the origin and center chains at the fold turn. Unlike a symmetric hairpin, the two chains are antiparallel and are offset by approximately 2.4 Å along the crystallographic *c*-axis,^46^ such that *R*_*i*–1_ and *R*_*i*+1_ experience different steric and packing constraints despite their equivalent distance from *R*_*i*_.

For the **3mer** models (Figure 9a), the most frequently sampled pucker transition pathway features a ^1^S_5_ pucker at *R*_*i*_ with ^3^S_1_ at *R*_*i*+1_, while the second most probable pathway features a ^5^S_1_ state at *R*_*i*_ and ^4^C_1_ at *R*_*i*+1_. The third most probable pathway is notable in that all residues remain in the canonical ^4^C_1_ conformation. For the **5mer** models (Figure 9b), the dominant pathways are characterized by boat conformations at *R*_*i*_, with ^1,4^B and B_1,4_ features in the first and second most probable pathways respectively, both with ^4^C_1_ at all other positions. Notably, the third and fourth most probable pathways both associates ^1^C_4_ pucker at *R*_*i*–1_ paired with ^1^S_5_ and ^1,4^B at *R*_*i*_, respectively. This is consistent with the initial puckering assignments, in which ^1^C_4_ at *R*_*i*–1_ is present in the low-energy **5mer-I** and **5mer-II** models but absent across all **3mer** models (Table 1), suggesting that the ^1^C_4_ conformation at *R*_*i*–1_ represents a stable configuration accessible to the 5-residue turn geometry but not to the 3-residue turn.

Across both models, the comparable widths of the major pathways in the Sankey diagrams indicate that multiple geometrically distinct puckering sequences along the three central residues at folded turn (*R*_*i*–1_, *R*_*i*_, *R*_*i*+1_) coexist with similar frequencies, rather than a single dominant pathway prevailing. This suggests that the chain-fold structure does not enforce a unique puckering solution but is instead compatible with a set of conformational channels of comparable energetic cost. This interpretation is consistent with the earlier observation that several fold models converge to similar puckering distributions despite different initial conformations. This suggests that these conformational channels represent intrinsically stable configurations of the fold turn rather than artifacts of the initial setup. While structural adjustment appears to remain localized within three residues of the turn, the broader conformational landscape and multiple accessible puckering pathways associated with the 5-residue turn suggest that this geometry may nevertheless serve as a kinetic intermediate during chain folding or lattice reorganization.

## Conclusions

This study presents a systematic computational investigation of chain-folded cellulose-II fibrils, examining the structural consequences of incorporating 3-residue (**3mer**) and 5-residue (**5mer**) folded fragments into an idealized 6 × 6 cellulose-II crystal model. The simulated models suggest that chain folding may be structurally compatible with a preserved cellulose-II lattice. The crystalline core retains its fundamental unit cell parameters, XRD patterns, hydrogen bonding network, and canonical ^4^C_1_ ring conformation throughout 300 ns of MD simulation, consistent with the possibility that the presence of chain folds does not compromise the bulk crystalline integrity of the fibril.

The structural perturbation introduced by chain folding is localized to the chain-fold ends, exhibiting elevated RMSD values, reduced hydrogen bond density and significant pyranose ring distortion relative to both the crystalline core and the unfolded **CM** reference. These features are interconnected, as the geometric constraints of the fold turn necessitate the adoption of high-energy boat and skew-boat conformations at the turn residue *R*_*i*_, which in turn drives a conformational redistribution of the hydroxymethyl group from the canonical *gt* rotamer toward *gg* and minor *tg* states. This shift simultaneously relieves steric strain and enables noncanonical intrachain hydrogen bonding contacts that partially compensate for the loss of the interchain interlayer hydrogen bonding network characteristic to the cellulose-II crystal. We note that no evidence of significant fibril twisting was observed in either the **CM** or chain fold cellulose-II models over the course of the simulations.

Previous studies of single layer folded chain cellulose-II structure modeled with the COMPASS force field^18^ showed that the chain-fold region preferentially adopted skew-boat conformations. In contrast, using a GLYCAM06/TIP3P potential, both the **3mer** and **5mer** models could sample a broader range of noncanonical puckering states. Specifically, the **3mer** turn imposed a more confined conformational penalty, with pyranose ring pucker distortion largely restricted to *R*_*i*_, while a non-negligible population of residues retained the canonical ^4^C_1_ conformation even at the turn residue. The **5mer** turn model, by contrast, distributed ring strain more broadly, with noncanonical puckering states including ^1^C_4_ and B_O,3_ extending to the adjacent *R*_*i*–1_ residue. Despite these differences in the specific puckers sampled, both studies agree in confining ring distortion to the fold turn region, and this agreement across two independent force fields suggests that the localization reported here is likely to be reproducible with alternative carbohydrate force fields, rather than an artifact specific to GLYCAM06/TIP3P.

In both folded chain models, the fold turn is compatible with a set of stable conformational channels, with the **5mer** turn model affording greater conformational flexibility. This enabled access to a broader range of puckering combinations than the more geometrically constrained **3mer** turn model. Nevertheless, the distortion is confined within the three central residues *R*_*i*–1_, *R*_*i*_ and *R*_*i*+1_, thus, should such folded structures exist, the 3-residue turn may represent a more realistic fold architecture for cellulose-II. However, we emphasize that these findings are necessarily tentative. The models presented here are idealized constructs intended to assess structural feasibility of folded systems in aqueous solution; we have not modeled the folding process nor considered the effect on this of environmental factors such as alkaline treatment and viscose processing conditions. Quantifying the free-energy landscape and kinetic feasibility of chain-fold formation would be a valuable extension of this work, for which replica-exchange molecular dynamics may be a suitable approach. Further experimental validation remains key to determine whether such chain-folded configurations are realized in native or processed cellulose-II systems.

The notion that cellulose may be chain folded is one that is worth revisiting. Being able to contain more disordered material at the ends of the chains could aid the processing of cellulose, particularly by hydrolysis to less polydisperse nanocrystals. It is also important to note that folds in cellulose chains may not exist ubiquitously throughout most sources of the material, except when synthesized in constrained situations, or when the molecular weight is sufficiently high. In primitive algae and bacterial sources this has been shown to occur, but higher plants appear to produce extended chain conformations, although folds may be possible if high molecular weight chains are confined. These relationships are worthy of further work on the subject, and may also lead to applications as well as being of fundamental interest.

## Supplementary Material



## Notes

Additional details of the simulated systems used for molecular dynamics calculations are provided, as well as computed X-ray parameters, and a discussion of sampling of ring pucker by **3mer** and **5mer** replicas (PDF)
