# Supplementary material for: Molecular Dynamics Simulations Suggest Folded Chain Ends Compatible with the Cellulose-II Fibril Structure
Source: Biomacromolecules. 2026 Aug 5;27(9):6086–95. doi: 10.1021/acs.biomac.6c00983 (PMC13580024; doi:10.1021/acs.biomac.6c00983)
Supplement: Supplementary file 1 [file bm6c00983_si_001.pdf]

Supporting Information

# Molecular dynamics simulations suggest folded chain ends compatible with the cellulose-II fibril structure

*Linghan Kong[1], Stephen J. Eichhorn[2,3]*, Richard A. Bryce[1]**

1. Division of Pharmacy and Optometry, University of Manchester, Oxford Road, Manchester, M13 9PT, United Kingdom

2. Bristol Composites Institute, School of Civil, Aerospace and Design Engineering, Faculty of Science and Engineering, University of Bristol, BS8 1TR, United Kingdom.

3. School of Chemistry, Cantock's Close, University of Bristol, Bristol BS8 1TS, United Kingdom.

**Table S1** Summary of simulation systems.

| System | Folded turn model | Chain arrangement | No. of residues per chain | Total no. of glucose residues | Simulation water box (Å) | No. of simulation replicates | Total simulation length (ns) |
|---|---|---|---|---|---|---|---|
| ref | - | 36 individual chains in a 6 × 6 antiparallel arrangement | 40 | 1440 | 100 × 100 × 250 | 3 | 3 × 300 |
| 3mer | I, II, III, IV, V | 6 folded chains in a 1 × 6 arrangement; each folded chain comprises 6 antiparallel segments linked by 5 folded turns | 245 | 1470 | 100 × 100 × 250 | 15 (3 per folded turn model) | 15 × 300 |
| 5mer | I, II, III, IV, V | 6 folded chains in a 1 × 6 arrangement; each folded chain comprises 6 antiparallel segments linked by 5 folded turns | 245 | 1470 | 100 × 100 × 250 | 15 (3 per folded turn model) | 15 × 300 |

**Table S2** Comparison of peak positions (2θ) and normalized relative intensities of the characteristic cellulose-II Bragg reflections for the folded-chain model and ideal cellulose-II reference.

| Plane | Ideal cellulose-II 2θ | 3mer-I-a 2θ | Ideal cellulose-II intensity | 3mer-I-a intensity |
|---|---|---|---|---|
| (1-10) | 12.20° | 11.83° | 0.28 | 0.52 |
| (110) | 19.95° | 19.98° | 1.0 | 1.0 |
| (020) | 22.10° | 22.03° | 0.92 | 0.90 |

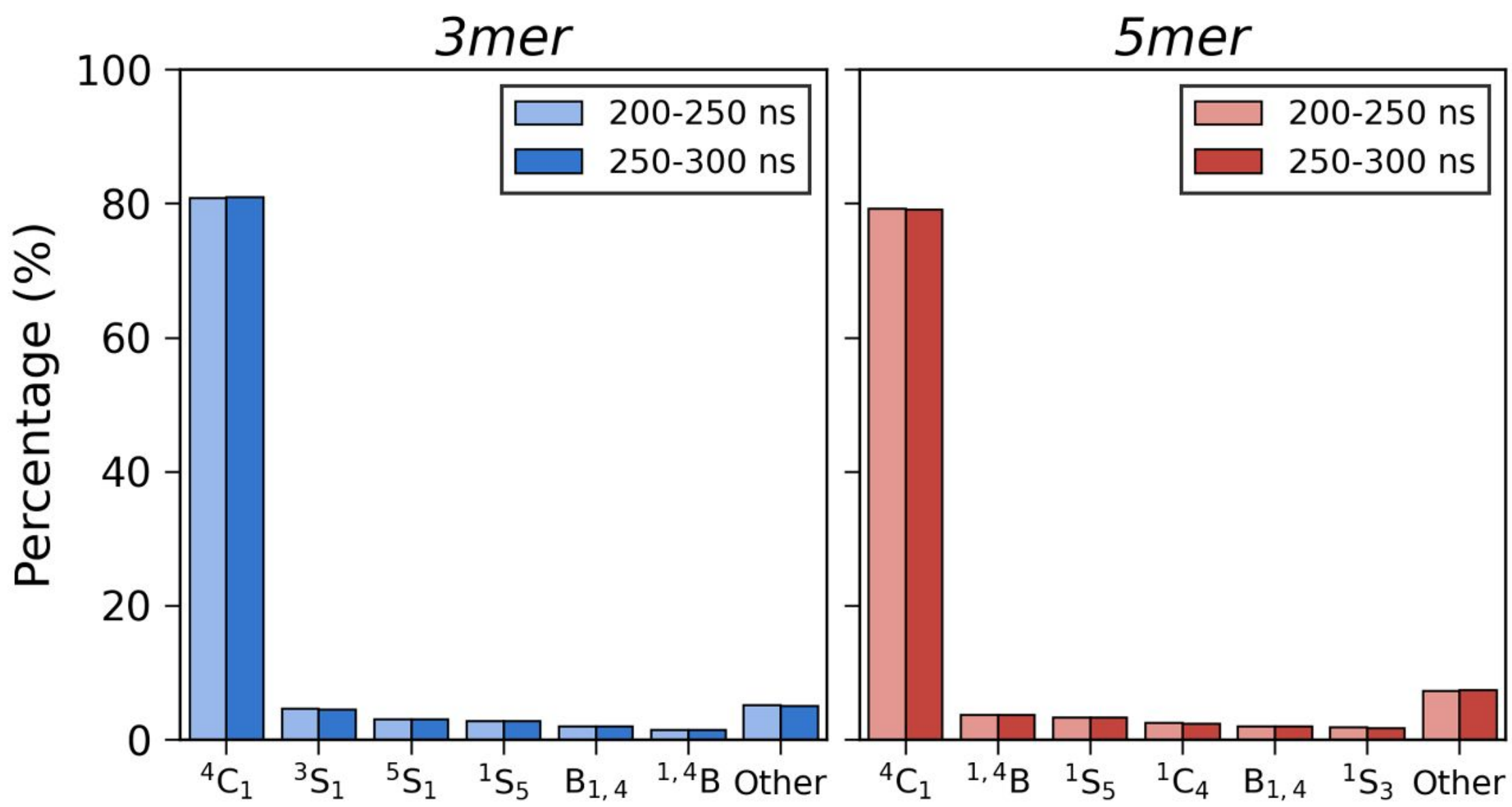


**Figure S1** Percentage of the dominant ring pucker states for the **3mer** (left) and **5mer** (right) models, within the chain ends region. The distribution is compared over two consecutive 50 ns windows.

## Sampling of ring pucker by 3mer and 5mer replicas

For the **3mer** models, **3mer-I** and **3mer-II**, initialized with $^5S_1$ and $B_{1,4}$ at the central fold residue $R_i$ respectively (Figure 1a), both exhibit similar overall puckering profiles dominated by $^5S_1$, $B_{1,4}$ and $^3S_1$ states (Figure 8). **3mer-III**, despite being initialized with

a different puckering combination of $^{O}S_2$ at $R_{i-1}$ and $^{2}S_O$ at $R_i$, converges to an identical distribution to that of **3mer-I** and **3mer-II** (Figure 1a). In contrast, **3mer-IV** and **3mer-V**, initialized with $^{1}S_5$ and $^{1,4}B$ at $R_i$ respectively, retain these conformations throughout the trajectories, despite their higher GFN2-xTB conformational energy of 8.9 and 11.6 kcal/mol relative to **3mer-I** (Table 1), suggesting a different stable state.

A similar pattern is observed for the **5mer** models. **5mer-I** and **5mer-II**, both initialized with a $^{1}C_4$ pucker at $R_{i-1}$ and $^{1}S_5$/$^{1,4}B$ at $R_i$, converge to nearly identical puckering distributions, with the $^{1}C_4$ pucker retained at $R_{i-1}$ throughout the trajectories (Figure 8). **5mer-III**, initialized in the $^{1,4}B$ state at $R_i$ but $^{4}C_1$ at $R_{i-1}$, yields a different distribution relative to **5mer-II**, and also differs from the similarly puckered **3mer-III** model, likely reflecting differences in spatial orientations and steric interactions arising from the crystal packing environment. **5mer-IV** shares the same $R_{i-1}$ to $R_{i+1}$ puckering combination as **3mer-I**, with the exception of $^{2}S_O$ replacing $^{4}C_1$ at $R_{i-1}$; however, this $^{2}S_O$ pucker is not retained and instead converges to the neighboring $B_{3,O}$ conformation; while the $^{5}S_1$ pucker at $R_i$ also transitions to the neighboring $B_{1,4}$ conformation. Similarly, **5mer-V** does not retain its initial $B_{3,O}$ pucker at $R_{i-1}$, with the $B_{1,4}$ pucker at $R_i$ exploring the neighboring $^{3}S_1$ and $^{5}S_1$ conformations.
